# Study on the Mechanical and Toughness Behavior of Epoxy Nano-Composites with Zero-Dimensional and Two-Dimensional Nano-Fillers

**DOI:** 10.3390/polym14173618

**Published:** 2022-09-01

**Authors:** Xiaodong Li, Qi Wang, Xu Cui, Xinwen Feng, Fei Teng, Mingyao Xu, Weiguo Su, Jun He

**Affiliations:** 1 College of Aerospace Engineering, Shenyang Aerospace University, Shenyang 110136, China; 2 College of Civil Aviation, Shenyang Aerospace University, Shenyang 110136, China; 3 Naval University of Engineering, National Key Laboratory of Science and Technology on Vessel Integrated Power System, Wuhan 430033, China

**Keywords:** nano-silica, Borazon, nanocomposite, mechanical strength, dynamic mechanical analysis

## Abstract

The mechanical properties of epoxy resin can be enhanced by adding nanofillers into its matrix. This study researches and compares the impacts of adding nanofillers with different dimensions, including two-dimensional boron nitride and zero-dimensional silica, on the mechanical and toughness properties of epoxy resin. At low fractions (0–2.0 wt%), 2DBN/epoxy composites have a higher Young’s modulus, fracture toughness and critical strain energy release rate compared to SiO_2_/epoxy composites. However, the workability deteriorated drastically for BN/epoxy composites above a specific nanofiller concentration (2.0–3.0 wt%). BN prevents crack growth by drawing and bridging. SiO_2_ enhances performance by deflecting the crack direction and forming voids. Additionally, the dimension and content of nanofiller also influence glass transition temperature and storage modulus significantly.

## 1. Introduction

In various areas, including the aerospace and aircraft industries, epoxy adhesives have been widely used [1,2,3,4], e.g., the adhesive bonding of fibers to form composites [5], and they can also be used to join things together [6]. Epoxy resins have not gained their popularity without basis. The reason why epoxy resin is widely used is its critical functional properties including adhesion, flame resistance, chemical and thermal stability [7,8,9]. A simple and effective way to improve the mechanical properties of epoxy resin adhesives is by incorporating reinforcing phases. Recently, various technologies including adding soft nanoparticles and rigid nanofillers have been developed to improve the brittleness and fracture energy of epoxy resins, such as SiC [10], alumina and carbon-based fillers [11]. It has been suggested that the dimensionality of nanofillers plays an essential role in improving their reinforcing effect on the structural and functional performance of polymer matrix nanocomposite materials [12,13]. Researchers prepared epoxy resin nanocomposite specimens containing 0.1 wt% nanomaterials, 2D graphene oxide and 3D nitrogen-doped graphene to improve the tensile strength of the resin; the values of tensile strength were improved by 16% and 19%, respectively [14,15]. The degree of reinforcement of 2D graphene and 3D-CNT to epoxy resin composites is also different. When the content is 1 wt%, the strengthening ratio is 20% and 50%, respectively [16]. The main reasons are that different nanofillers cause different properties. Certain dimensions of nanofillers exhibited the self-lubrication phenomenon as a result of which the workability did not deteriorate, however, the other dimensions of nanomaterials became entangled. Too much nanofiller drastically destroys the workability of epoxy composites because of van der Waals forces [12,14,16]. 

Silica nanoparticle (0D nano-SiO_2_) has been widely used for composites with enhanced performance [17,18]. For instance, Dong-Jun Kwon et al. [19] studied the effects of silica nanofillers on various mechanical properties and the thermal aging stability of GF/epoxy composites. The results show that the reinforcement effects of the two Si nanofillers are different due to the different bonding methods. The epoxy composites modified by SiC have better thermal aging resistance than epoxy-SiO_2_. Roopesh Kumar Sinha et al. [17] reported on the improvement of the dynamic behavior of fiber composite materials by silica nanofillers. Nano SiO_2_ also displays compatibility with the matrix’s polymer chains and high interfacial chemical reactivity with the fiber’s sizing [20]. Boron nitride is chemically represented as BN, a 2D nanoparticle. BN is a compound of boron and nitrogen and has excellent mechanical properties. It has the property of being a heat and chemically resistant refractory material. Nano BN generally exists in the form of a 2D crystalline material. Due to its stability and flexibility, it is widely used in aviation products [21]. Wattanakul et al. [22] found that the thermal conductivity of BN-filled epoxy composite increased with filler content over the concentration range 0–37 vol.%. It has been observed that the hardness of aluminium-boron nitride nanocomposite depends on the wt% of BN filler [23]. Aluminium-boron nitride nanocomposites have a better opportunity to be used as a heat transfer material in high-temperature applications [24]. Hence, comparing the use of silica and BN nanoparticles clarifies the influence of using inorganic nanomaterial for epoxy resin. 

The addition of different fillers to the polymer can affect a variety of physical properties of the composite. Bekeshev et al. [25] researched ochre as a modified additive (0.5 parts by mass) and a filler (75 parts by mass) of epoxy composition can improve the physical and mechanical properties of the complex. Ochre affects the structure formation process and the structure of epoxy composites, thus increasing its thermal, heat and fire resistance. Jamali et al. [26] studied the effects of GONP modification and S-GONPs loading on the mechanical properties of UBF/epoxy composite. The highest values in mechanical strengths were obtained at 0.4 wt% S-GONPs. The tensile, flexural, and compressive strengths of this specimen were 16%, 47%, and 51% greater, respectively, than that of UBF/epoxy composite. Mostovoy et al. [27] researched modification of the epoxy composition affects the physical and mechanical properties, processes of the structure formation, morphology, and thermal stability of reinforced epoxy composites. Long et al. [28] researched the addition of SiO_2_ nanoparticles treated with inactive groups, NH_2_ active groups, and C_4_H_8_ active groups to epoxy resins that could improve the mechanical properties and bonding properties of epoxy adhesives.

At present, some scholars have studied the improvement of the properties of epoxy resin by nanofillers. These studies show that different kinds and different forms of nanofillers have different effects on the properties of epoxy resin. However, it is not common to compare SiO_2_ and BN with different components. The focus of this study is to compare the effects of different dimensional nanofillers such as two-dimensional (2D) boron nitride (BN) and zero-dimensional (0D) silicon dioxide (SiO_2_) on the mechanical properties and toughness of epoxy resin. Studying of the different strengthening effects and mechanisms of SiO_2_ and BN on epoxy resin can expand the idea of epoxy resin modification and make it more convenient for these composites to be applied in practical production. Moreover, it is important to determine unambiguously how silica and BN nanofillers influence the fracture behaviors of epoxy. This article clarifies and understands the complicated enhancement between the different dimension fillers on toughening epoxy. In this article, specimens of pure epoxy and nanocomposites are reinforced with 2D and 0D inorganic nanomaterials. This paper studied and compared the impact of adding SiO_2_ and BN to the epoxy-based adhesive on its lap shear strength, toughness and dynamic mechanical behaviors in the range of loading 0–3 wt%. The mechanism of action of different dimensional materials in epoxy resin was revealed.

## 2. Materials and Methods

### 2.1. Materials

Nano silica (Nano-poxF400) as a colloidal solution (40 wt%) has been dispersed in epoxy resin by Hanse Chemie AG, Geesthacht, Germany. The diameter is 20–23 nanometers, and the shape is spherical. Nano hexagonal crystal BN (30–50 nm in size, purity > 99.9%) was purchased as a solid from Shanghai Chaowei Nanotechnology Co., Ltd., Shanghai, China. Epoxy resin, diglycidyl ether of bisphenol A (DGEBA, Araldite-F) with an epoxide equivalent weight of 182–196 g/equiv, and the viscosity is 11,000~14,000 mPa.s, was supplied by Ciba-Geigy, Australia. Hardener Jeffamine D230 (denoted J230) was kindly provided by Huntsman, China. The basic physical properties of BN and SiO_2_ is shown in Table 1. The content of each component of different epoxy composites samples is shown in Table 2.

### 2.2. Preparation of Composite

**Figure 1:**
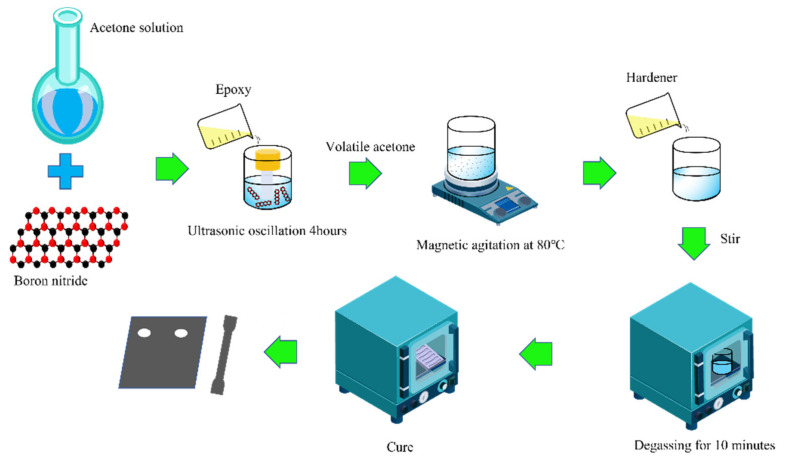
The preparation process of epoxy/BN composites.

In this study, two epoxy composites were prepared: (i) epoxy/silica nanoparticle (SiO_2_) composite adhesive (ii) epoxy/BN nanosheets composite adhesive. The fabrication of the composite adhesive was carried out as follows. Each epoxy resin was mechanically mixed with a pre-weighed amount of BN nanoparticle to produce a composite adhesive with a fraction range of 1.0–3.0 wt%. Ultrasonic oscillation was a simple and effective method to uniformly disperse nanomaterials in epoxy resin. The epoxy resin mixed with the nanofiller is ultrasonically shaken to make the nanofiller uniformly dispersed. Magnetic stirring at 80 °C effectively removed acetone and obtained a resin matrix uniformly dispersed with nanofillers. A calculated quantity of J230 (weight ratio of DGEBA/J230 = 3.3:1) was added to the mixture. Each mixture was carefully degassed in a vacuum oven for 10 min to remove bubbles and the mixture was poured into molds. A two-stage curing process was then carried out: 80 °C for 2 h and then 120 °C for 10 h. Both sides of the samples were polished with sandpaper until all visible marks had disappeared. The samples were then thermally treated at 120 °C for 120 min to reduce the defects caused by polishing. The preparation process of epoxy/BN composites is shown in Figure 1. The preparation process of the epoxy/SiO_2_ composite was similar to that of the epoxy/BN composite. 

**Figure 2:**
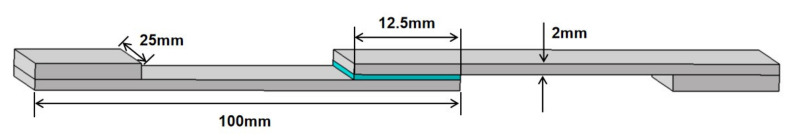
The structure of the single lap bonded specimen.

The lap shear strength (LSS) test measures the joint strength by dividing the failure load by adhesive bond area. According to ASTM D5573–99, the structure of the single lap bonded specimen is shown in Figure 2, and the substrate material used is aluminum alloy. Two kinds of epoxy resin adhesives mentioned are used as adhesives and treated according to the same curing process.

### 2.3. Characterizations

Dynamic mechanical analysis (DMA) of nanocomposites was performed using the DMA 2980, TA Instruments (USA). Single cantilever clamped rectangle samples had tests conducted at a frequency of 1 Hz. The temperature range was 20−150 °C, and the heating rate was 5 °C/min. The dimensions of the samples were 35 mm × 10 mm × 3.5 mm. Dumbbell samples were used for static tensile tests at a speed of 0.5 mm/min. ISO13586 standard indicated that compact tension (CT) can test fracture toughness (K_ΙC_) of epoxy nanomaterials. The dimensions of the CT samples were 30 mm × 30 mm × (5–6) mm. Each component was tested at least three times and the average was recorded. Scanning electron microscopy (SEM) was carried out to observe the fracture surfaces (crack tip and propagation zone) of compact tension (CT) specimens using SEM, ZEISS Sigma 300 (ZEISS, Oberkochen, Germany). A thin layer of platinum was used to coat the fractured surface and then examined at an accelerating voltage of 10 kV.

## 3. Results

### 3.1. Fracture Toughness and Mechanical Properties 

**Figure 3:**
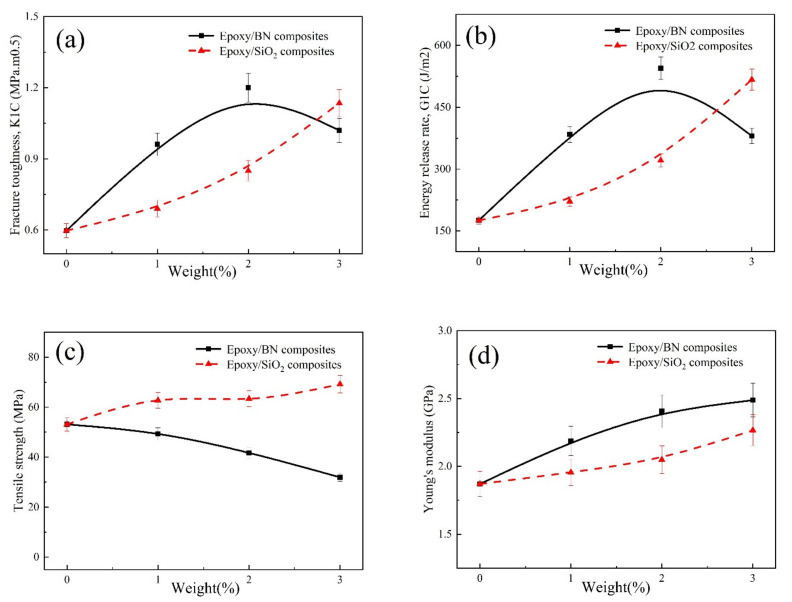
(**a**) Fracture toughness, (**b**) Critical strain energy release rate, (**c**) Tensile strength, (**d**) Young’s moduli of epoxy/SiO_2_ and epoxy/BN composites.

Mechanical properties of epoxy resin after incorporating SiO_2_ and BN nanoparticles are plotted in Figure 3 as a function of filler amount; namely, fracture toughness (K_ΙC_), critical strain energy release rate (G_ΙC_), Young’s modulus and tensile strength. K_ΙC_, which measures the absorbed energy to propagate sharp crack, and G_ΙC_ are primary ways to evaluate the toughness of an epoxy nanocomposite. With the changes to the two nanofiller components, Figure 3a,b shows the rules of both fracture toughness (K_ΙC_) and critical strain energy release rate (G_ΙC_) of epoxy composites. Both K_ΙC_ and G_ΙC_ of epoxy nanocomposites are obviously enhanced by adding BN and SiO_2_ up to 2.0 wt% at different increments. For instance, at 2.0 wt% filler, the K_ΙC_ and G_ΙC_ of epoxy/SiO_2_ composites are improved by 42% and 83%, respectively, while there was a 101% and 210% increase in the case of BN. The increase in attributes of nanocomposites will slowly decrease once the BN is increased to 2.0 wt%; at 3.0 wt%, K_ΙC_ and G_ΙC_ the benefits begin to decrease but are still higher than that of neat epoxy. In contrast, the performance growth of epoxy/SiO_2_ is stable and sustainable. At 3.0 wt% nano-0DSiO_2_, K_IC_ and G_ΙC_ of nanocomposites are enhanced by 90% and 195%.

**Figure 4:**
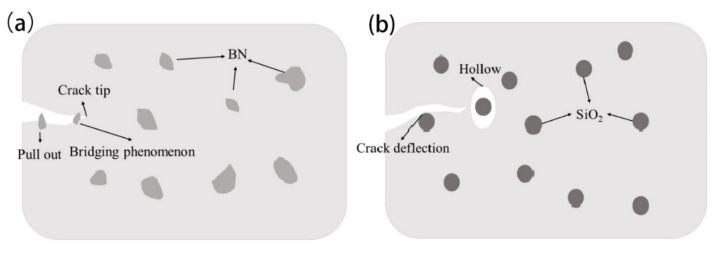
Schematic diagram of nanofiller reinforcement mechanism, (**a**) BN reinforced composite, (**b**) SiO_2_ reinforced composite.

Figure 3c,d shows Young’s moduli and tensile strengths of epoxy/BN and epoxy/SiO_2_ composites. With the addition of BN and SiO_2_, Young’s modulus will continue and slowly increase, respectively. Nevertheless, the 2D materials BN had a better effect on the improvement of Young’s moduli of the epoxy compound. The reason is that BN can absorb more energy when the epoxy compound fractures as shown in Figure 4. When the content of nanofiller increases, the tensile strengths of epoxy/BN will slowly decrease. Epoxy resin is a brittle cross-linked polymer, and the debonding and stiffening effect between polymer and nanofillers will cause its tensile strength to decrease. The 0D nano-SiO_2_ exhibited a special self-lubrication phenomenon, as a result of which the workability did not deteriorate [12,14]. With an increase of nanofiller content, tensile strengths of epoxy/SiO_2_ nanocomposites steadily increase. This conforms with the previous studies [6,29,30].

Figure 3 shows the different rules of BN and SiO_2_ for resin composites when the content of the filler is beyond 2.0 wt%. The enhancement value of SiO_2_ exceeds BN in all behaviors tested including K_ΙC_, G_ΙC_ and Young’s modulus. Among them, the tensile strength decreases slowly due to the addition of BN. The details of the properties of epoxy composites are given in Table 3 and Table 4. For example, at 3.0 wt%, the K_ΙC_, G_ΙC_, Young’s modulus and tensile strength of epoxy/SiO_2_ are 1.135 MPa.m^0.5^, 517.11 J/m^2^, 2.27 GPa and 69.18 MPa compared to 1.020 MPa.m^0.5^, 380.53 J/m^2^, 2.49 GPa and 31.94 MPa in epoxy/BN composites, respectively.

The reasons for the results are as follows. At low content (0–2 wt%), there is enough space for nanomaterials to be dispersed. They strengthen the resin matrix and improve stress transfer and load distribution between the epoxy matrix and nanofillers. While at a high fraction, because of the 2D structure of BN, the phenomenon of aggregation and clustering will appear earlier. These aggregated nanofillers formed defects, caused stress concentration and accelerated crack propagation. The addition of 2D and 0D nanomaterials will have different effects on the properties of epoxy composites, the reason is as follows:

(i) In some sections of epoxy/BN nanocomposites, interlayer van der Waals forces lead to the accumulation and irregular dispersion of BN nanosheets, causing a reduction in the monotonous load transfer from resin to nanomaterials. This process occurs less frequently in the SiO_2_/epoxy due to the structure of SiO_2_ nanomaterials.
(ii) Compared with zero-dimensional silica, BN with two-dimensional structure can form better mechanical interlocking with epoxy resin and improve the mechanical proper-ties of the composite.

### 3.2. Lap Shear Strength of the Adhesive Joints

**Figure 5:**
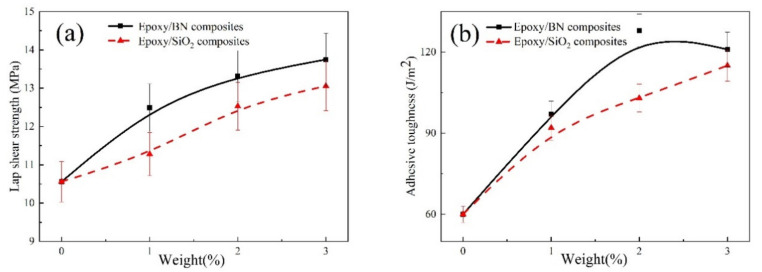
(**a**) Lap shear strength and (**b**) DCB toughness of epoxy/SiO_2_ and epoxy/BN composite adhesives.

Figure 5a compares the LSS values of epoxy/SiO_2_ composite adhesives to 2D dimensional epoxy/BN composite adhesives. It is clear that the two nanomaterials enhance the LSS of the epoxy resin at all fractions (<3.0 wt%), which is attributed to the intrinsic outstanding strength of nanomaterials; nanomaterials generally reinforce polymeric materials. For example, at 3.0 wt%, SiO_2_ increased the epoxy’s LSS by 29% and BN increased the epoxy’s LSS by 30%. The addition of nanofillers can improve the interface performance of resin and aluminum, and thus nanofillers can enhance single lap shear strength [31,32].

Moreover, these results indicate that the epoxy/BN nanocomposite had a higher LSS than the epoxy/SiO_2_ nanocomposite. The reason is that compared with spherical SiO2, sheet BN with two-dimensional structure can form better mechanical interlocking with epoxy resin, resulting in higher lap shear strength of the sample. Specifically, epoxy/BN nanocomposite showed 10%, 6%, and 5% increases in LSS at fraction levels of 1, 2, and 3 wt%, respectively, when compared to the epoxy/SiO_2_ nanocomposite. With the addition of BN and SiO_2_, the LSS steadily increases, but the growth curve of the epoxy/BN composites gradually flattens as the amount of nanofiller increases. The reason for this is that the 2D nanofillers became entangled, and the workability deteriorates as the content of nanofiller is increased.

Specimens under loading I, double cantilever beam (DCB) can have their delamination toughness calculated [33]. The adhesive toughness of epoxy composite adhesives was tested using DCB samples and plotted in Figure 5b. Adding SiO_2_ and BN to epoxy adhesives enhances the adhesive fracture toughness. For example, the adhesive toughness of epoxy/3wt%2DBN reached 121 J/m^2^ while it was 115 J/m^2^ for an epoxy/SiO_2_ composite adhesive. It confirms the results obtained for bulk composites in Figure 3.

Figure 5b shows the influence of BN on the epoxy resin is different to that of SiO_2_ with the increase of nanofiller content. The main reason is the high concentration of BN in the epoxy resin which probably led to its agglomeration.

### 3.3. Dynamic Mechanical Analysis (DMA)

Generally, we determined loss modulus, storage modulus, glass transition temperature (T_g_) and damping factor based on DMA. These properties are tested by studying the behavior of composites under different frequencies, stresses and temperatures [34,35]. For inorganic filler/polymer composites, viscoelasticity can reflect the molecular relaxation and interaction between components [36,37].

**Figure 6:**
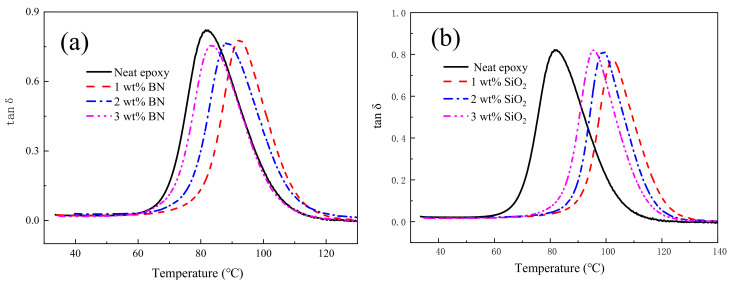
Tan delta for (**a**) epoxy/BN and (**b**) epoxy/SiO_2_ composites.

The damping ratio (tan δ) is driven by the ratio of loss and storage modulus. It gives the equilibrium between the elastic and viscous phase. The peak value of tan δ determines the value of T_g_ [38]. The tan δ of epoxy composite as a function of temperature is plotted in Figure 6. Table 5 records the T_g_ values of epoxy nanocomposites. T_g_ is obviously enhanced at lower wt% of BN and SiO_2_; such as, at 1 wt%, T_g_ increases from 83 °C (neat epoxy) to 92 °C when BN is added, and to 102 °C in the case of using SiO_2_. When more nanofiller (>1 wt%) is added to epoxy, T_g_ steadily declines.

**Figure 7:**
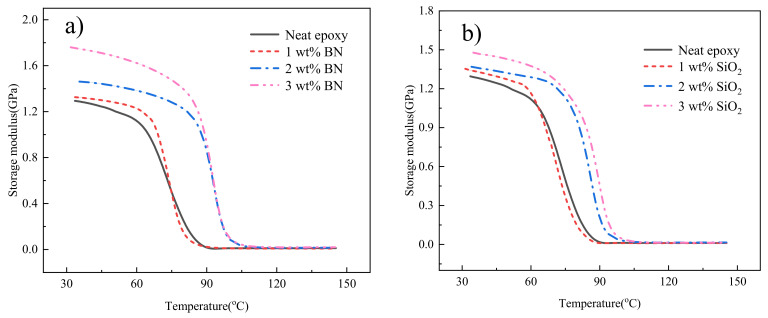
Storage modulus for (**a**) epoxy/BN and (**b**) epoxy/SiO_2_ composites.

Figure 7 shows the changing trend of the storage modulus of composite with nanofiller dimension and content. The temperature range is 30 °C to 150 °C. The role of storage modulus is similar to that of Young’s modulus, and both are attributes that indicate the stiffness of the composite [39]. The storage modulus of the two nano-material-reinforced epoxy composites show similar changes. As the filler content increases, the storage modulus continues to increase. Among them, BN has a better enhancement effect.

The reason for the improved storage modulus is that the evenly dispersed nanofillers are entangled with epoxy resin, which improves the resistance of the composite material to mechanical deformation. Simultaneously, the filler increases the crosslink density, restricts the fluidity of the epoxy resin chain, leading to increased stiffness. As the temperature increases (exceeding the glass transition temperature), the composite transforms into a viscoelastic, the fluidity increases, and the storage modulus decreases.

### 3.4. Morphology of Fracture Surfaces and Mechanism

The mechanical properties shown at macroscopic level can be examined at microscopic level. Here, we researched the fracture toughening mechanism of nanocomposites by studying the rough fracture surface of CT specimens using SEM analysis. The SEM micrographs show the dispersion and toughening of nanofillers.

**Figure 8:**
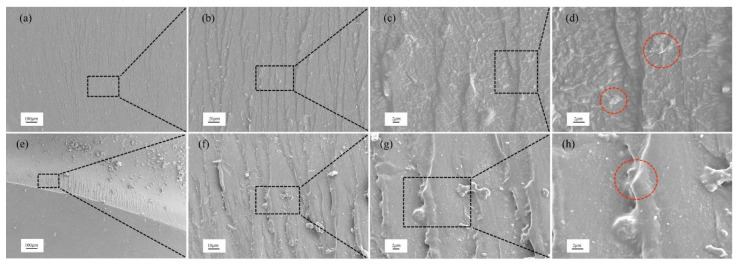
(**a**–**d**) epoxy/SiO_2_ composite and (**e**–**h**) epoxy/BN composite at 1 wt%.

Figure 8a–h shows the fractography of epoxy/SiO_2_ and epoxy/BN nanocomposites with different magnifications at 1.0 wt%. The image shows that SiO_2_ has good dispersibility, and the red mark in Figure 8d shows that SiO_2_ has a positive effect on the fracture of epoxy resin. Figure 8e–h shows the same effect for epoxy/BN composites. 

**Figure 9:**
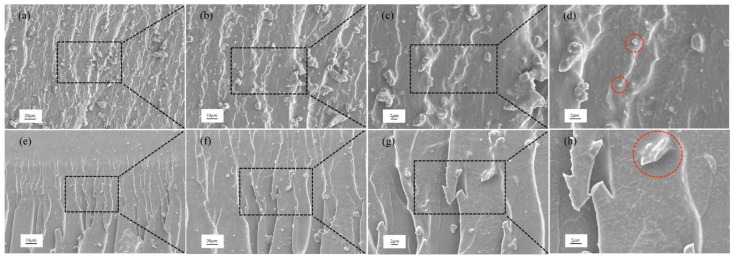
(**a**–**d**) epoxy/SiO_2_ composite and (**e**–**h**) epoxy/BN composite at 3 wt%.

Figure 9a–h display the fracture morphology of epoxy nanocomposites from high to low magnification at 3.0 wt%. As indicated by the mark, the entanglement and agglomeration of nanomaterials are evident at 3.0 wt%. The entangled BN can be considered a defect. Nevertheless, as shown in Figure 8d, SiO_2_ delays entanglement due to its 0D shape. The main reason is that the nanomaterials with this structure exhibited the self-lubrication phenomenon [12].

The continuous rough surface indicates the energy being absorbed by the crack propagating to a fracture [34]. However, the reinforcement mechanism of the two fillers is not the same. Figure 4 is a schematic diagram of the reinforcement principle of two nanofillers. To clearly express, the actual situation is appropriately enlarged and exaggerated. As shown in Figure 4a, BN is a two-dimensional structural filler, formed from a mechanical interlock with epoxy. When cracks are initiated and propagated in the composite material, BN pulls out or forms a bridging phenomenon in the resin. Figure 4b shows the difference between SiO_2_. SiO_2_ is a rigid nanomaterial. When the crack encounters SiO_2_ during the propagation process, it will be forced to deflect. In addition, when the resin matrix undergoes plastic deformation, the rigid SiO_2_ does not participate in the deformation. Therefore, SiO_2_ can form hollows in the resin matrix to enhance the toughness of the composite material.

In short, with a low content, BN can absorb more energy by its 2D shape to further enhance the mechanical capabilities of the nanocomposite. At high content, SiO_2_ delays entanglement and agglomeration by its self-lubricating ability and its properties are vitally enhanced.

## 4. Conclusions

In this paper, the mechanical properties and dynamic thermomechanical analysis of 2D and 0D inorganic-nanofiller/epoxy nanocomposites were researched. These results revealed that BN and SiO_2_ can improve the mechanical behaviors of the nanocomposites. 

However, BN and SiO_2_ have different enhancement mechanisms due to different nanostructures. They improved the capabilities of epoxy nanocomposites at different increments. For example, at 2.0 wt%, the K_ΙC_, G_ΙC_, Young’s modulus and tensile strength of epoxy/SiO_2_ was 0.85 MPa.m^0.5^, 320 J/m^2^, 2.05 GPa and 63 MPa compared to 1.20 MPa.m^0.5^, 544 J/m^2^, 2.41 GPa and 41 MPa in epoxy/BN composites, respectively. At 3 wt%, K_ΙC_ and G_IC_ of epoxy/BN started to decrease but were still higher than that of neat epoxy. 

Additionally, as the content of the two nanofillers changes, the glass transition temperature and storage modulus of the composite change. Among them, the changing trend of the storage modulus is similar to that of Young’s modulus, that is, it increases steadily with the increase of filler content. The reason is that the addition of nanofillers enhances the stiffness of the composite. The glass transition temperature reaches the maximum when the filler mass ratio is 1 wt%. Corresponding to BN and SiO_2_ reinforced composite, the maximum glass transition temperatures are 92.51 °C and 102.06 °C, respectively.

SEM images of the rough fracture surface revealed the role of nanomaterials in the process of crack growth and specimen fracture. At a low fraction, BN and SiO_2_ strengthen the resin matrix through their respective structures and methods. At a high fraction, agglomeration of nanofillers began to occur. Due to the self-lubricating phenomenon of SiO_2_, the agglomeration phenomenon will be delayed. Epoxy composites with nanofillers withstand higher loads. It better transmits loads as a matrix for fiber composites or fiber metal laminates.

Compared with BN, SiO_2_ is less likely to agglomerate at low fractions. This is be-cause of the larger van der Waals forces between the layers of the 2D sheet BN, which makes it easier to stick together.

## Figures and Tables

**Table 1:** The basic physical properties of BN and SiO_2_.

Materials	Color and Form	Relative Molecular Mass	Relative Density	Particle Size(nm)	Particle Morphology
SiO_2_	white powder	60.08	2.31	20–23	spherical
BN	white powder	24.82	2.26	30–50	Graphite-type layered structure (hexagonal structure)

**Table 2:** The content of each component in different composite samples.

Composite Sample	Epoxy (g)	J230 (g)	40%SiO_2_/Epoxy (g)	BN (g)
Pure epoxy composite	46.05	13.95	0	0
1 wt% Epoxy/BN composite	45.59	13.81	0	0.6
2 wt% Epoxy/BN composite	45.13	13.67	0	1.2
3 wt% Epoxy/BN composite	44.67	13.53	0	1.8
1 wt% Epoxy/SiO2 composite	44.90	13.60	1.5	0
2 wt% Epoxy/SiO2 composite	43.74	13.26	3	0
3 wt% Epoxy/SiO2 composite	42.59	12.91	4.5	0

**Table 3:** Toughness and mechanical properties of epoxy/BN composites.

Fraction of BN Nanoparticle (wt%)	Tensile Strength (MPa)	Young’s Modulus (GPa)	$\mathrm{Fracture}\;\mathrm{Toughness},\;K_{Ιc}\;(\mathrm{MPa}.m^{0.5})$	$\mathrm{Energy}\;\mathrm{Release}\;\mathrm{Rate},\;G_{Ιc}(J/m^{2})$
0(neat epoxy)	53.12 ± 2.66	1.87 ± 0.09	0.597 ± 0.03	175.48 ± 8.77
1	49.29 ± 2.48	2.19 ± 0.11	0.961 ± 0.04	384.10 ± 19.21
2	41.69 ± 2.08	2.41 ± 0.12	1.200 ± 0.06	544.53 ± 27.23
3	31.94 ± 1.60	2.49 ± 0.12	1.020 ± 0.05	380.53 ± 19.03

**Table 4:** Toughness and mechanical properties of epoxy/SiO_2_ composites.

Fraction of SiO_2_ Nanoparticle (wt%)	Tensile Strength (MPa)	Young’s Modulus (GPa)	$\mathrm{Fracture}\;\mathrm{Toughness},\;K_{Ιc}\;(\mathrm{MPa}.m^{0.5})$	$\mathrm{Energy}\;\mathrm{Release}\;\mathrm{Rate},\;G_{Ιc}(J/m^{2})$
0(neat epoxy)	53.12 ± 2.66	1.87 ± 0.09	0.597 ± 0.03	175.48 ± 8.77
1	62.74 ± 3.14	1.96 ± 0.09	0.690 ± 0.003	221.61 ± 11.08
2	63.36 ± 3.17	2.05 ± 0.10	0.850 ± 0.04	320.88 ± 16.04
3	69.18 ± 3.46	2.27 ± 0.11	1.135 ± 0.06	517.11 ± 25.86

**Table 5:** Glass transition temperature of neat epoxy and its composites.

Epoxy Composite (wt%)	Epoxy/BN Composites		Epoxy/SiO_2_ Composites	
	$T_{g}$	Increment (%)	$T_{g}$	Increment (%)
Neat epoxy	83.29	-	83.29	-
1	92.51	11.07	102.06	22.54
2	88.08	5.75	98.83	18.66
3	83.35	0.07	95.50	14.66

## Data Availability

The data that supports the findings of this study are available within the article.
